# Patient perceptions of glucocorticoids in anti-neutrophil cytoplasmic antibody-associated vasculitis

**DOI:** 10.1007/s00296-017-3855-6

**Published:** 2017-11-09

**Authors:** Joanna C. Robson, Jill Dawson, Peter F. Cronholm, Susan Ashdown, Ebony Easley, Katherine S. Kellom, Don Gebhart, Georgia Lanier, Nataliya Milman, Jacqueline Peck, Raashid A. Luqmani, Judy A. Shea, Gunnar Tomasson, Peter A. Merkel

**Affiliations:** 1 Faculty of Health and Applied Sciences, University of the West of England, Bristol, UK; 2 School of Clinical Sciences, University of Bristol, Bristol, BS2 8HW UK; 3 Nuffield Department of Population Health, University of Oxford, Oxford, UK; 4 Department of Family Medicine and Community Health, University of Pennsylvania, Philadelphia, PA USA; 5 Oxfordshire, UK; 6 Department of Family Medicine and Community Health, Mixed Methods Research Laboratory, University of Pennsylvania, Philadelphia, PA USA; 7 PolicyLab, Children’s Hospital of Philadelphia, Philadelphia, US USA; 8 Columbus, OH USA; 9 Boston, MA USA; 10 Department of Rheumatology, University of Ottawa, Ottawa, Canada; 11 Nuffield Department of Orthopaedics, Rheumatology and Musculoskeletal Sciences, University of Oxford, Oxford, UK; 12 School of Medicine, University of Pennsylvania, Philadelphia, PA USA; 13 Department of Public Health Sciences, University of Iceland, Reykjavik, Iceland; 14 Division of Rheumatology, Departments of Medicine and Epidemiology, University of Pennsylvania, Philadelphia, PA USA

**Keywords:** ANCA-associated vasculitis, Glucocorticoids, Patient perspectives, Granulomatosis with polyangiitis (Wegener’s), Eosinophilic granulomatosis with polyangiitis, Microscopic polyangiitis

## Abstract

Granulomatosis with polyangiitis (GPA), microscopic polyangiitis (MPA), and eosinophilic granulomatosis with polyangiitis (EGPA) are multisystem diseases of small blood vessels, collectively known as the anti-neutrophil cytoplasmic antibody (ANCA)-associated vasculitides (AAV). This study explores the patient’s perspective on the use of glucocorticoids, which are still a mainstay of treatment in AAV. Patients with AAV from the UK, USA, and Canada were interviewed, using purposive sampling to include a range of disease manifestations and demographics. The project steering committee, including patient partners, designed the interview prompts and cues about AAV, its treatment, and impact on health-related quality of life. Interviews were transcribed and analysed to establish themes grounded in the data. A treatment-related code was used to focus analysis of salient themes related to glucocorticoid therapy. Fifty interviews were conducted. Individual themes related to therapy with glucocorticoids emerged from the data and were analysed. Three overarching themes emerged: (1) Glucocorticoids are effective at the time of diagnosis and during relapse, and withdrawal can potentiate a flare, (2) glucocorticoids are associated with salient emotional, physical, and social effects (depression, anxiety, irritation, weight gain and change in appearance, diabetes mellitus, effect on family and work); and (3) patient perceptions of balancing the risks and benefits of glucocorticoids. Patients identified the positive aspects of treatment with glucocorticoids; they are fast-acting and effective, but, they voiced concerns about adverse effects and the uncertainty of the dose-reduction process. These results may be informative in the development of novel glucocorticoid-sparing regimens.

## Introduction

The anti-neutrophil cytoplasmic antibody (ANCA)-associated vasculitides (AAV) comprise patients with granulomatosis with polyangiitis (GPA), microscopic polyangiitis (MPA) and eosinophilic granulomatosis with polyangiitis (EGPA) (AAV) [1]. The AAVs are multisystem life- and organ-threatening diseases with renal, respiratory, ear nose and throat, dermatological and neurological involvement [2–5]. Almost 40% of patients will suffer a disease relapse [6], and all patients suffer mental and physical health impairments at diagnosis [7], and during follow-up [2, 8, 9]. Patients rate psychological and social aspects of the disease higher than their physicians do [10].

Glucocorticoid use, in combination with immunosuppressive therapy, is the cornerstone of management in AAV; high doses are used during induction followed by a slow dose-reduction, aiming for glucocorticoid-free remission [11–13]. Increased rates of diabetes mellitus, fractures, gastrointestinal bleeding, hypertension, infection, and cataract have been attributed to glucocorticoid therapy in AAV [14, 15], with the duration of glucocorticoid therapy associated with high levels of irreversible damage [16]. Weight gain, sleep disturbance, lipodystrophy, and neuropsychiatric disturbances (including irritability, anxiety, depression, and hyperactivity) have also been reported [17, 18].

Despite the ubiquity of glucocorticoid therapy, there has not been a systematic investigation of the patient experience of their use in AAV. An awareness of patients’ treatment experiences and preferences is essential to guide the development of new therapeutic regimens, improve shared patient-doctor decision making [19] and facilitate patient compliance with prescribed therapy [20]. We interviewed patients with AAV from the United Kingdom (UK), United States (US), and Canada (CA) about their disease and the impact of being treated with glucocorticoids on their Health-Related Quality of Life (HRQoL).

## Patients and methods

Patients with AAV were recruited from three rheumatology centres in the UK, US, and Canada. Inclusion criteria were: age ≥ 18, ability to give informed consent, and a diagnosis of GPA, MPA, or EGPA. Patients were purposively sampled to include all three diseases, chronicity of disease (diagnosis or flare < 2 years prior to enrolment or > 2 years), age, sex, and organ involvement (Table 1).

**Table 1:** Demographic data of 50 patients with ANCA-associated vasculitis interviewed for this study

Demographics	Oxford (*n* = 18)	Ottawa (*n* = 14)	Philadelphia (*n* = 17)	Total
**Diagnosis**				
GPA	8	8	9	26
MPA	3	1	3	7
EGPA	7	6	5	18
**Sex**				
Male	12	6	6	24
Female	6	9	11	27
**Age**				
< 50	3	2	5	11
≥ 50	15	13	12	40
**Diagnosis or flare**				
< 2 years	13	5	16	34
≥ 2 years	5	10	1	17
**Organ involvement**				
Kidney	9	2	5	16
Lung	12	12	10	34
ENT	10	14	11	35
Neuro	4	1	6	12

### Data collection

The Steering Committee, including four patients from the UK and US (SA, JP, GL and DG), methodologists and clinician researchers, defined a set of neutral, non-directive interview prompts and cues on the patient experience with, and impact on HRQoL of, AAV and its treatment (Table 2). Participants’ responses were probed as appropriate. Interviews were performed by a qualitative researcher in the US (KK) and two clinical research fellows in the UK (JR) and Canada (NM). Study approvals were given by the NHS Research Committee South West-Central Bristol (12/SW/0252), Ottawa Hospital’s Research Ethics Board (20120604-01H) and the University of Pennsylvania Office of Regulatory Affairs (817899).

**Table 2:** Interview prompts and cues in relation to treatment

**Treatment specific prompts and cues**
Describe how the treatment for vasculitis affected you
Good/bad affects
Timing of effects
Effects other than physical
Effect on day to day life
**Other prompts and cues that elicited responses related to glucocorticoids**
Diagnosis
Tell me about the time when you first got your diagnosis
Describe how getting the diagnosis of vasculitis affected you
Disease flares
How would you describe “having a flare”?
Describe what you notice physically when your symptoms start to flare
Describe what you notice psychologically when your symptoms start to flare
Over what period of time does the flare develop?
Tell me about any clues you get that the vasculitis is about to, or is starting to, flare up
Tell me about what do you do when you think a flare is starting
What helps?
What makes it worse?
What thoughts do you have about future flares?

## Analysis

Interviews continued until no new substantive themes arose indicating ‘data saturation’ and to satisfy the sampling framework [21]. Data was organised using NVivo version 10 [22]. All interviews were transcribed and inductive analysis was used to identify salient themes in relation to glucocorticoids [23]. A treatment-related code was used to identify all references to the experience of treatment. With input from the Steering committee patient partners, themes were then categorised into three overarching themes.

## Results

**Fig. 1:**
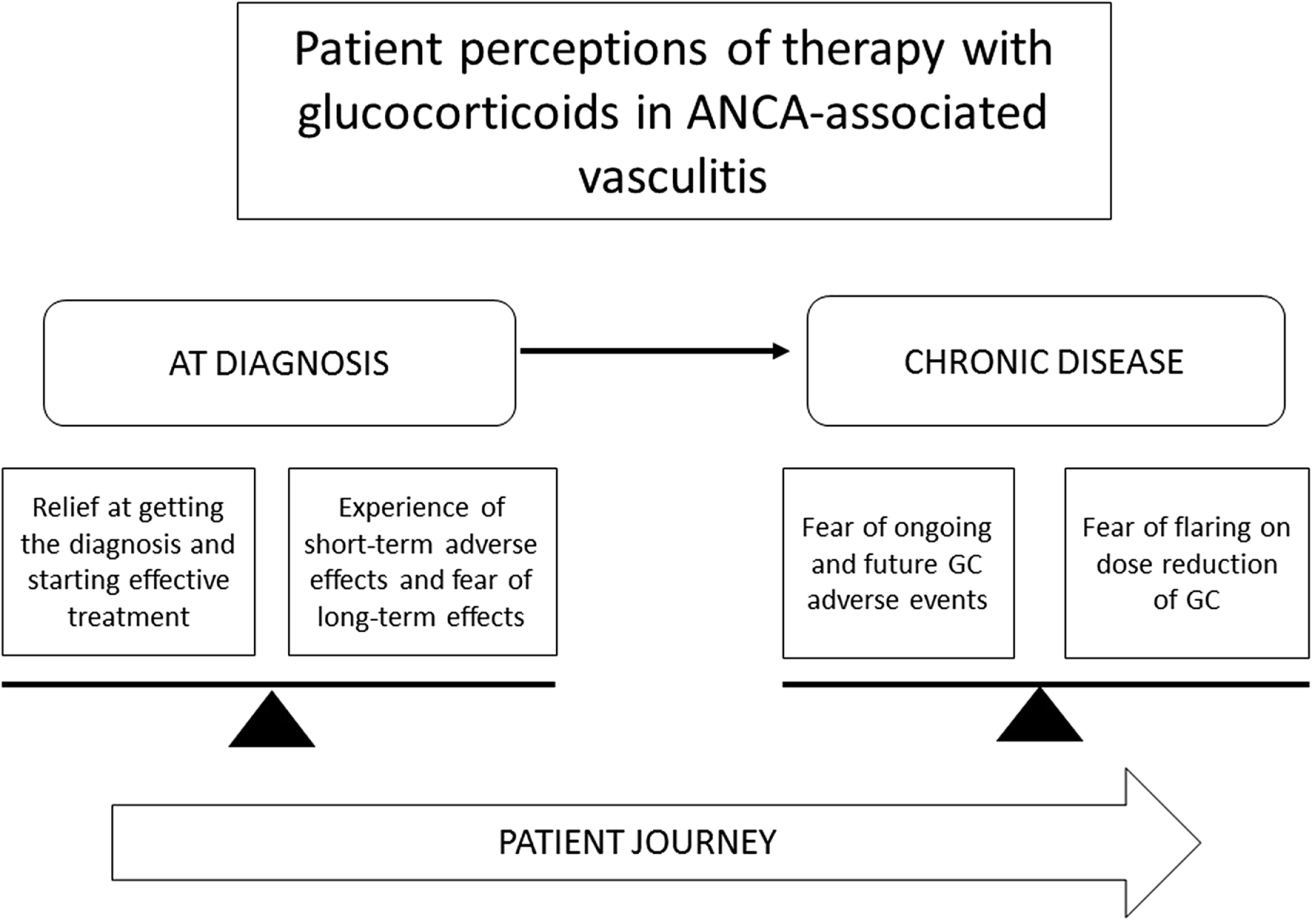
Patient experience of therapy with glucocorticoids in the treatment of ANCA-associated vasculitis: the balance between an effective treatment and concern about adverse effects over time

We undertook semi-structured individual interviews with 50 patients with AAV; demographics of the study patients are shown in Table 2. Forty individual themes related to therapy with glucocorticoids emerged from the data, these were then grouped into 13 categories and three overarching themes: (1) Glucocorticoids are effective and withdrawal can potentiate a flare of AAV, (2) salient emotional, physical and social effects of glucocorticoid therapy, and (3) balancing the pros and cons of glucocorticoids (see Table 3 and Fig. 1).

**Table 3:** Individual and overarching patient themes related to glucocorticoid therapy

Individual theme name	Individual theme definition
**Theme A. Glucocorticoids are effective and withdrawal can potentiate a flare**	
Positive effects of GC	Relief at starting treatment after prolonged investigations.
	Symptoms improve quickly; feeling of the problem being fixed.
	Staying on GC “for ever” may control symptoms
Flaring on reduction of glucocorticoids	Withdrawal symptoms on reducing GCs Flare linked to reducing GCs
	Feeling of GC reduction being an “experiment”
	Increasing dosages of GCs resolving flares swiftly
**Theme B. Description of salient emotional, physical and social effects of glucocorticoid therapy**	
Change in appearance	Significant weight gain
	Puffiness and change in facial features
	Not recognising oneself
	Feeling “weird”
	Not able to wear certain clothes
	Feeling depressed by change in appearance
Others comments on appearance	Partners comments about weight
	Family support to lose weight
	Family unsupportive or derogatory about weight
	Others unable to understand increase in energy with GCs
	Looking “well” on GC masking underlying illness to others
Appetite	Hunger pains and increase in appetite
Adaptations needed to reduce weight gain	Exercise and diet
Diabetes	New diabetes and difficult to control established diabetes
	Monitoring of blood sugars: painful and intrusive
Skin changes	Easy bruising and skin fragility
Sleep disturbance	Difficulty getting to sleep/insomnia
	Requiring less sleep
Muscle strength	Reduced muscle strength
Emotional symptoms	Feeling manic or euphoric
	Increased energy/hyperactivity
	Anxiety
	Mood swings
	Depression
	Effect of personality change on personal life and or work
**Theme C. Patient perceptions of balancing the risks and benefits of glucocorticoids**	
Process of weighing up use of GC	Long-term side effects versus short-term death
	Body can only take GC for so long
	Maintenance dose may be needed long-term
Concern about long-term adverse effects	Bone loss
	Diabetes
	Glaucoma

### Theme 1

Glucocorticoids are effective and withdrawal can potentiate a flare of ANCA-associated vasculitis.

At the point of diagnosis, patients reportedly felt relief that the treatment was starting and that glucocorticoids were fast-acting and effective: “Well, I guess it was important that I was embarking on some treatment. You know, particularly with the high-dose steroids, you know, I really felt good (71-year-old female, EGPA, US)”; and “I came in here on Friday. I could not walk. I started the steroids on Saturday and on Sunday I was walking down the ward (69-year-old male, GPA, UK)”.

Difficulties with stopping glucocorticoids were commonly reported: “every time we change the prednisone it is a little bit like going through withdrawal (52-year-old female, GPA, Canada)”. Patients also described the unpredictable nature of glucocorticoid reduction: “it was clearly unknown what was going to happen if I tried to get off of it······it was just an experiment. I mean, I think it was done with the full understanding that if it did not work, I was ready to go back on the steroids. I mean, and that’s exactly what happened (71-year-old male, EGPA, US)”.

### Theme 2

Salient emotional, social and physical effects of glucocorticoid therapy.

### Emotional effects

Participants reported abnormally high-energy levels on glucocorticoids: “the treatment then started with very high dose steroids. That was interesting because I was, to use a term, wired. I mean, it’s known that high-dose steroids almost make you manic, or certainly euphoric (71-year-old male, EGPA, US)”. This affected sleeping patterns: “I would have incredible energy levels, require very little sleep. You know, I’d go to sleep for a couple of hours and then wake up and got involved with all kinds of projects around the house (71-year-old male, EGPA, US)”, and “I find I can’t sleep through the night and I think I blame that for my fatigue (55-year-old male, MPA, UK)”.

Others reported depression, anxiety, and irritability: “when my steroids are high… I’m taking, well for me say about 30 mg a day, I get a kind of a depression almost. And it’s almost like-Well why should I bother getting up? (53-year-old female, EGPA, UK)”, and “I mean, prednisone doesn’t help with anxiety. It definitely makes you more on edge and snappish and—especially when you’re going up and down, so I’m trying to keep it together (76-year-old male, EGPA, Canada)”. Social interactions could be affected: “···in my personal life. Probably in my work life···I would find myself going into extremely dark holes. I’d start an argument, and go down an argument that I knew that was complete stupid argument, but couldn’t back off of it, and then have to go… So all that was all part of, of, of when I got these flare-ups, and then the constant treatment of steroids (51-year-old male, EGPA, UK)”.

### Weight gain and change in appearance

Hunger and increased appetite were widely reported: “it had a huge effect. Everything changed. I was taking a lot of prednisone so—I don’t know if you’ve ever been on prednisone, but you get these crazy hunger pains. It’s like you just—there’s like this feeling you need to satiate with food all the time (68-year-old female, EGPA, Canada).”

Both women and men described a change in appearance and weight gain with glucocorticoids, which had a range of consequences: “as I started gaining the weight, like my size went up, I’ve been a size six for my entire life, and then all of a sudden last summer, when I went to get my summer clothes, nothing was fitting me and it was just like I was always worried about my weight, because I’m such a small person. And it kind of bummed me out a little bit, because it was everywhere, like upper arms and then like my shoulders puffed up and I just, my face, especially, because you look at yourself in the mirror every day. And I didn’t like what I was seeing, it just wasn’t me, it makes you feel weird about yourself (67-year-old female, GPA, UK)”, and “because I’ve always been slim, always. I work in the catering industry. And I always wore the nice dinner suits, nicely cut. And all of a sudden, well, forget about the suits. I couldn’t find shirts for my neck, because my neck was too big for the shirts. And it was just… I think that did more damage, in my mind, than the whole thing (49-year-old male, GPA, UK)”. For some participants weight gain impacted interpersonal relationships: “at one point while I was still on high-dose prednisone my boyfriend said something about how he could still see me on the inside. Like I had gained so much weight at that point it was ridiculous (35-year-old female, GPA, US)”. Self-management strategies for avoiding weight gain were described: “I still don’t like taking steroids, and I don’t want to gain all that weight. But I’ve read books on how to not put as much weight on, and I don’t give into the hunger pains anymore (35-year-old female, GPA, US).

Changes in appearance could make the AAV more visible, leading to unwanted attention: “I hadn’t told them about it and then they see me and my one niece is a nurse, so she automatically knew I was on steroids, because they saw my face and they were like, wow, what’s going on (57-year-old female, GPA, Canada)”. Whereas some found glucocorticoids masked how unwell they were, so others were less understanding: “Everybody thinks I’m fine. I’ve got steroids and everything to, to push me up. But they forget that we’ve got the inside as well. The inside is not so good (49-year-old male, GPA, UK)”.

### Other physical effects

Other physical adverse events reported included new-onset or worsening of diabetes mellitus: “and then I had to check my blood sugar in the morning and at night, which I never had to do before in my life and watch what I eat and it’s just like geez, and it just seemed like it was all at once (52-year-old female, GPA, Canada)”; skin fragility: “my skin is like tissue paper. I’m cutting the grass, I brush against a shrub and look down—I’m bleeding like a pig (64-year-old male, GPA, Canada)”, and muscle weakness: “I just don’t have the muscle power in my legs. And also my arms—I don’t have the muscle power in my arms. That’s been there from the very start. But it seems to worsen when I’m on the steroids (53-year-old female, EGPA, UK)”.

#### Theme 3

Patient perceptions of balancing the risks and benefits of glucocorticoids.

Patients often reflected on the potential consequences of long-term treatment with glucocorticoids: “apparently, according to the doctors, if I don’t get off the prednisone soon, I’m gonna be in a world of trouble. Because it has—well, it has a lot of side effects. Really bad side effects. Bone loss, glaucoma, diabetes, just nasty (32-year-old female, GPA, US)”. There was often a feeling of time running out in terms of how long glucocorticoids could safely be continued in the face of continued flaring of disease: “My worst worry is about that prednisone, because right now it is not too bad, I am not too old, but I am pretty sure I am not able to use 25 mg of prednisone for the rest of my life, because something else is going to go (47-year-old male, EGPA, Canada)”.

Patients spoke about balancing short-term benefits versus long-term risks: “You like what it’s doing for you, as far as pain management, but then, the side effects are like.., that’s why the goal right now is to eventually, I have to be off of it, because your body can only take it for so long (58-year-old female, GPA, US)”. Patients balanced similar risks and benefits with medications other than glucocorticoids for the treatment of AAV: “I know that some of the medications have got negative long-term side effects, but its negative long-term versus short-term death (64-year-old male, GPA, Canada)”. The balance of needing an effective treatment, but having concerns about adverse effects is shown in Fig. 1.

However, as patients became more experienced in their disease and its treatment, some were able to pragmatically weigh-up the options: “I’d love to be off the steroids completely and maybe that will happen eventually. But I think you know from my experience at the moment, maybe a maintenance dose is the way to go (53-year-old female, EGPA, UK)”.

## Discussion

Glucocorticoids are a key part of induction therapy in AAV [11, 24–26]. This study documents the wide range of perceptions patients have about their use of these powerful medications. Patients can be positive about treatment with glucocorticoids at diagnosis and during disease flares, and perceive their use as fast and effective. These opinions are consistent with those previously reported by patients with rheumatoid arthritis [27]. The ideal glucocorticoid dose-reduction regimen in AAV has not yet been determined [11] with longer courses potentially reduce the relapse rate but increasing the risk of infection [28, 29]. Patients in this study were aware of the uncertainty of the glucocorticoid dose-reduction process and raised this as a specific concern, particularly in terms of unpredictable risk of flaring.

Patients have insight into the risks and benefits of glucocorticoids, as demonstrated in this study and elsewhere [27], and this insight can be used in shared doctor–patient decision making, including pragmatic decisions about dose reduction or maintenance at low doses for which the evidence of adverse effects is not clear-cut [13, 30]. Previous qualitative research has identified that patients with AAV would value receiving more education about their disease [31], with patients rating information on treatment as extremely important [31, 32]. This study and research in rheumatoid arthritis has identified that patients commonly have concerns about the safety of glucocorticoids [33]; sometimes focusing on rare but serious complications, without knowledge of the overall risk of events [27]. Addressing these educational needs for patients with AAV, including putting adverse effects and benefits into context, may help reduce unnecessary anxiety about the use of glucocorticoids, increase adherence to therapy, and improve patient awareness of practical steps that can be taken to reduce adverse events [34, 35].

A wide range of physical, social and emotional adverse effects related to use of glucocorticoids were articulated by patients in this study. The emotional aspects were particularly highlighted, as per previous ranking exercises [10] and quantitative studies of patients with AAV in general, e.g., a questionnaire study of 51 patients found depression in 25% and anxiety in 40% [9]. Attribution of psychological manifestations to disease or treatment has been attempted, for example, higher pain Visual Analogue Scores (VAS) and Hospital Anxiety and Depression Scores (HADs), and lower SF-36 social function, and SF-36 energy/vitality scores have been reported in patients with AAV taking ≥ 10 mg/day of oral prednisolone (*n* = 8) compared to < 10 mg/day (*n* = 42) [36], although this analysis was not adjusted for disease activity. Patient descriptions of the order in which events occur, can bring insights into the causal pathway of certain symptoms. One example concerns patients’ descriptions of significant weight gain and change in appearance, leading to a loss of sense of self, negative changes in the behaviour of people around them, and then low mood. Whilst it is likely that the cause of depression is multifactorial in AAV, these patients’ descriptions may help target future interventions to reduce the psychological impact of AAV and its treatment.

Fatigue is a principal component of impaired HRQoL in AAV [2] and is associated with a number of biopsychological factors, including sleep disturbance [37]. The link between high-dose glucocorticoids and insomnia, potentially exacerbating fatigue, was proposed by patients within this study and may represent a modifiable determinant of fatigue in a subset of patients. Irritability and anger secondary to glucocorticoids were also highlighted as impacting family and work colleagues; a US study did not find an overall difference in HRQoL among spouses of those with stable AAV, compared to US norms [38]. Physical effects reported in this study included weight gain, diabetes mellitus, and skin fragility. Other potential factors, for example infection or cardiovascular disease [14, 15], were not reported, possibly because they were not experienced by the patients within the sample, or patients did not attribute these complications to glucocorticoids.

This is the largest qualitative study of patients with AAV, and the first to describe the patient experience of glucocorticoids. Patients were purposively sampled to ensure involvement of patients with a spectrum of disease presentations and demographic features. One limitation of this analysis is potential confounding by indication, i.e., patients with more severe disease may be treated with higher dosages of glucocorticoids. Descriptions given by patients of the timing of specific symptoms are helpful to determine the causal pathway of flare, increased glucocorticoid use and adverse effects. Another potential limitation was that patients with severe disease may have been less likely to participate in interviews; to reduce this occurence, patients on dialysis and those receiving induction chemotherapy were purposively sampled to ensure inclusion in the sample. We were concerned that patients might have felt uncomfortable making negative comments about medication regimens in interviews with clinician researchers in the UK and Canada, compared to the non-clinical qualitative researcher in the US. There is no indication of this from the high number of negative comments received from UK and Canadian patients about glucocorticoids, but this effect cannot be completely excluded.

Alternatives to prolonged courses of high-dose glucocorticoids, including the use of reduced-dose regimens and the development of novel glucocorticoid-sparing agents, is a priority for the vasculitis research community [16] and patients alike [31]. Patient perceptions of the benefits of glucocorticoids, i.e., these medications work quickly at diagnosis and flare, may be helpful in terms of defining success in the search for alternative regimens with a reduced side effect profile. When assessing novel therapies and regimens, it will be important to accurately capture the relevant range of physical, emotional, and social symptoms and treatment side effects, and how these interact with patients’ function and HRQoL. A disease-specific PRO instrument incorporating potential glucocorticoid-related benefits and losses is vital to allow a comprehensive assessment of the impact of a new treatment in relation to therapy.

## References

[1] Jennette JC, Falk RJ, Bacon PA, Basu N, Cid MC, Ferrario F (2013). 2012 revised International Chapel Hill Consensus Conference Nomenclature of Vasculitides. Arthritis Rheum.

[2] Basu N, Jones GT, Fluck N, MacDonald AG, Pang D, Dospinescu P (2010). Fatigue: a principal contributor to impaired quality of life in ANCA-associated vasculitis. Rheumatology (Oxford).

[3] Comarmond C, Pagnoux C, Khellaf M, Cordier JF, Hamidou M, Viallard JF (2013). Eosinophilic granulomatosis with polyangiitis (Churg-Strauss): clinical characteristics and long-term followup of the 383 patients enrolled in the French Vasculitis Study Group cohort. Arthritis Rheum.

[4] Stone JH (2003). Limited versus severe Wegener’s granulomatosis: baseline data on patients in the Wegener’s granulomatosis etanercept trial. Arthritis Rheum.

[5] Lhote F, Cohen P, Genereau T, Gayraud M, Guillevin L (1996). Microscopic polyangiitis: clinical aspects and treatment. Ann Med Interne (Paris).

[6] Walsh M, Flossmann O, Berden A, Westman K, Hoglund P, Stegeman C (2012). Risk factors for relapse of antineutrophil cytoplasmic antibody-associated vasculitis. Arthritis Rheum.

[7] Walsh M, Mukhtyar C, Mahr A, Herlyn K, Luqmani R, Merkel PA (2011). Health related quality of life in patients with newly diagnosed anti-neutrophil cytoplasm antibody associated vasculitis. Arthritis Care Res.

[8] Tomasson G, Boers M, Walsh M, LaValley M, Cuthbertson D, Carette S (2012). Assessment of health-related quality of life as an outcome measure in granulomatosis with polyangiitis (Wegener’s). Arthritis Care Res.

[9] Koutantji M, Harrold E, Lane SE, Pearce S, Watts RA, Scott DG (2003). Investigation of quality of life, mood, pain, disability, and disease status in primary systemic vasculitis. Arthritis Rheum.

[10] Herlyn K, Hellmich B, Seo P, Merkel PA (2010). Patient-reported outcome assessment in vasculitis may provide important data and a unique perspective. Arthritis Care Res.

[11] Ntatsaki E, Carruthers D, Chakravarty K, D’Cruz D, Harper L, Jayne D (2014). BSR and BHPR guideline for the management of adults with ANCA-associated vasculitis. Rheumatology (Oxford).

[12] Mukhtyar C, Guillevin L, Cid MC, Dasgupta B, de Groot K, Gross W (2009). EULAR recommendations for the management of primary small and medium vessel vasculitis. Ann Rheum Dis.

[13] Groh M, Pagnoux C, Baldini C, Bel E, Bottero P, Cottin V (2015). Eosinophilic granulomatosis with polyangiitis (Churg-Strauss) (EGPA) consensus task force recommendations for evaluation and management. Eur J Intern Med.

[14] Robson J, Doll H, Suppiah R, Flossmann O, Harper L, Hoglund P (2015). Damage in the anca-associated vasculitides: long-term data from the European vasculitis study group (EUVAS) therapeutic trials. Ann Rheum Dis.

[15] Little MA, Nightingale P, Verburgh CA, Hauser T, De Groot K, Savage C (2010). Early mortality in systemic vasculitis: relative contribution of adverse events and active vasculitis. Ann Rheum Dis.

[16] Robson J, Doll H, Suppiah R, Flossmann O, Harper L, Hoglund P (2015). Glucocorticoid treatment and damage in the anti-neutrophil cytoplasm antibody-associated vasculitides: long-term data from the European Vasculitis Study Group trials. Rheumatology.

[17] McDonough AK, Curtis JR, Saag KG (2008). The epidemiology of glucocorticoid-associated adverse events. Curr Opin Rheumatol.

[18] Fardet L, Flahault A, Kettaneh A, Tiev KP, Genereau T, Toledano C (2007). Corticosteroid-induced clinical adverse events: frequency, risk factors and patient’s opinion. Br J Dermatol.

[19] Bowling A, Ebrahim S (2001). Measuring patients’ preferences for treatment and perceptions of risk. Qual Health Care.

[20] Stevenson FA, Cox K, Britten N, Dundar Y (2004). A systematic review of the research on communication between patients and health care professionals about medicines: the consequences for concordance. Health Expect.

[21] Kerr C, Nixon A, Wild D (2010). Assessing and demonstrating data saturation in qualitative inquiry supporting patient-reported outcomes research. Expert Rev Pharmacoecon Outcomes Res.

[22] International Q (2012). NVivo qualitative data analysis software Version 10.

[23] Pope C, Ziebland S, Mays N (2000). Qualitative research in health care: analysing qualitative data. BMJ.

[24] Jones RB, Tervaert JW, Hauser T, Luqmani R, Morgan MD, Peh CA (2010). Rituximab versus cyclophosphamide in ANCA-associated renal vasculitis. N Engl J Med.

[25] Stone JH, Merkel PA, Spiera R, Seo P, Langford CA, Hoffman GS (2010). Rituximab versus cyclophosphamide for ANCA-associated vasculitis. N Engl J Med.

[26] de Groot K, Harper L, Jayne DR, Flores Suarez LF, Gregorini G, Gross WL (2009). Pulse versus daily oral cyclophosphamide for induction of remission in antineutrophil cytoplasmic antibody-associated vasculitis: a randomized trial. Ann Intern Med.

[27] van der Goes MC, Jacobs JW, Boers M, Andrews T, Blom-Bakkers MA, Buttgereit F (2010). Patient and rheumatologist perspectives on glucocorticoids: an exercise to improve the implementation of the European League Against Rheumatism (EULAR) recommendations on the management of systemic glucocorticoid therapy in rheumatic diseases. Ann Rheum Dis.

[28] Walsh M, Merkel PA, Mahr A, Jayne D (2010). Effects of duration of glucocorticoid therapy on relapse rate in antineutrophil cytoplasmic antibody-associated vasculitis: a meta-analysis. Arthritis Care Res (Hoboken).

[29] McGregor JG, Hogan SL, Hu Y, Jennette CE, Falk RJ, Nachman PH (2012). Glucocorticoids and relapse and infection rates in anti-neutrophil cytoplasmic antibody disease. Clin J Am Soc Nephrol.

[30] Dixon WG, Bansback N (2012). Understanding the side effects of glucocorticoid therapy: shining a light on a drug everyone thinks they know. Ann Rheum Dis.

[31] Mooney J (2015) Research priority setting survey for Vasculitis UK In: Mills J, (ed) Vasculitis UK patient symposium

[32] Mooney J, Poland F, Spalding N, Scott DG, Watts RA (2013). In one ear and out the other—it’s a lot to take in: a qualitative study exploring the informational needs of patients with ANCA-associated vasculitis. Musculoskelet Care.

[33] Morrison E, Crosbie D, Capell HA (2003). Attitude of rheumatoid arthritis patients to treatment with oral corticosteroids. Rheumatology (Oxford).

[34] Duru N, van der Goes MC, Jacobs JW, Andrews T, Boers M, Buttgereit F (2013). EULAR evidence-based and consensus-based recommendations on the management of medium to high-dose glucocorticoid therapy in rheumatic diseases. Ann Rheum Dis.

[35] Liu D, Ahmet A, Ward L, Krishnamoorthy P, Mandelcorn ED, Leigh R (2013). A practical guide to the monitoring and management of the complications of systemic corticosteroid therapy. Allergy Asthma Clin Immunol.

[36] Herrmann C (1997). International experiences with the Hospital Anxiety and Depression Scale–a review of validation data and clinical results. J Psychosom Res.

[37] Basu N, McClean A, Harper L, Amft EN, Dhaun N, Luqmani RA (2013). Explaining fatigue in ANCA-associated vasculitis. Rheumatology.

[38] Carpenter DM, Thorpe CT, Lewis M, Devellis RF, Hogan SL (2009). Health-related quality of life for patients with vasculitis and their spouses. Arthritis Rheum.

